# A new heterandrous species of *Solanum* section *Gonatotrichum* Bitter (Solanaceae) from Bahia, Brazil

**DOI:** 10.3897/phytokeys.7.1855

**Published:** 2011-11-29

**Authors:** Leandro L. Giacomin, João Renato Stehmann

**Affiliations:** 1 ICB, Departamento de Botânica, Laboratório de Sistemática Vegetal, Universidade Federal de Minas Gerais – UFMG, Av. Antônio Carlos, 6627, Pampulha, Belo Horizonte, CEP 31270-901, MG, Brazil.

**Keywords:** heterandry, Caatinga, IUCN conservation status

## Abstract

A new species of *Solanum* from Brazil is described. *Solanum evolvuloides* Giacomin & Stehmann, **sp. nov.** belongs to section *Gonatotrichum*, a small group assigned to the Brevantherum Clade of the genus *Solanum*. It resembles *Solanum turneroides* Chodat, sharing with it heterandry, and *Solanum parcistrigosum* Bitter, with which it shares a similar habit and pubescence. Despite these similarities, the species can be recognized by its ovate-elliptic to cordiform leaf shape and more membranaceous leaf texture than the other species in the section, and stem, inflorescence axes, and calyx vestiture mainly composed of glandular hairs. *Solanum evolvuloides* is known to occur only in southeastern of Bahia state, Brazil, and in a preliminary assessment of the IUCN criteria can be considered a threatened species.

Resumo

Uma nova espécie de *Solanum* é descrita para o Brasil. *Solanum evolvuloides* Giacomin & Stehmann, **sp. nov.** é componente da seção *Gonatotrichum*, um pequeno grupo associado ao Clado Brevantherum. A espécie é similar a *Solanum turneroides*, com a qual compartilha a heterandria, e *Solanum parcistrigosum*, que por sua vez apresenta um hábito e indumento foliar semelhante. Apesar da similaridade, a espécie pode ser facilmente reconhecida pela forma da folha ovada-elíptica a cordiforme e pela texura mais membranácea que as outras espécies da seção, além do indumento do caule, eixo da inflorescência e cálice, constituído em sua maioria por tricomas glandulares pedicelados. *Solanum evolvuloides* tem sua distribuição conhecida somente para o sudeste do estado da Bahia, Brasil, e é indicada como uma espécie ameaçada de extinção.

## Introduction

*Solanum*, with about 1500 species, is one of the largest genera of flowering plants, and the largest genus within Solanaceae (Frodin 2004, Weese and Bohs 2007). The huge number of species and the complexity of its morphology have led several researchers to propose a considerable number of infrageneric classification systems during the past two centuries, not all of them congruent (e.g.: Sendtner 1846, Dunal 1852, Seithe 1962, D’Arcy 1991, Hunziker 2001). More recent use of molecular phylogenetic techniques has brought several modifications to past classifications and allowed a better understanding of the genus by elucidating several clades within it (Bohs 2005, Weese and Bohs 2007). One of the clades recognized in these studies was the Gonatotrichum clade that corresponds to *Solanum* sect. *Gonatotrichum* Bitter, a small section established in the beginning of the 20^th^ century (Bitter 1912). Species of the section are small herbs to shrubs mainly with a vestiture of unbranched (simple) hairs (except for *Solanum lignescens* Fernald), and have fruits with explosive dehiscence. Molecular studies have supported the maintenance of the section as a natural group, but they have shown that it is closely related to a morphologically quite different group, *Solanum* sect. *Brevantherum* Seithe, nested within the Brevantherum clade of Weese and Bohs (2007), mostly composed of shrubs to trees with stellate or echinoid hairs and fruits without explosive dehiscence. This curious close relationship between sects. *Gonatotrichum* and *Brevantherum* was not recognized in past classifications and has stimulated more in-depth studies in the section.

Section *Gonatotrichum* was treated by Nee (1989) as having only two species, but recent herbarium investigations and field work showed an underestimated diversity. A new species was recently described (*Solanum manabiense* S. Stern) and at that time the authors accepted five species in the section (Stern and Bohs 2009). Another new species was found by us, in the CEPEC herbarium (Centro de Pesquisas do Cacau, Bahia, Brazil), and is here described as *Solanum evolvuloides*. A detailed taxonomic study has reevaluated the circumscription and species limits of the section, and a complete revision of *Solanum* sect. *Gonatotrichum* is in preparation. This revision is part of a comprehensive species level taxonomic inventory of the genus (Knapp et al. 2004, http://www.solanaceaesource.org/).

## Materials and methods

We revised material in the following herbaria (acronyms from *Index Herbariorum*, http://sweetgum.nybg.org/ih/): BHCB, CEPEC, CESJ, CTES, CORD, ESA, ESAL, FUEL, HAS, HB, HUEFS, IAC, IAN, ICN, INPA, LPB, MBM, MBML, PACA, R, RB, SP, SPF, SPSF, UEC, UPCB, VIC, and WU. Images of types deposited in BR, MO, NY, P, and SI, kindly provided by the curators or available on collections websites, were also examined. Plants obtained in the field were cultivated in the greenhouse and fresh flowers were fixed in alcohol to permit detailed descriptions and illustrations. We assessed the conservation status using IUCN Red List Categories and Criteria (IUCN 2010).

## Taxonomic treatment

### Solanum evolvuloides

Giacomin & Stehmann sp. nov.

urn:lsid:ipni.org:names:77115891-1

http://species-id.net/wiki/Solanum_evolvuloides

Figs 1 2 

#### Latin.

Solano turneroidi *Chodat et* S. parcistrigoso *Bitter similis sed ab utroque foliis membranaceis ovato-ellipticis vel cordiformibus et pilis glanduliferis in caule, axibus inflorescentiae, et calyce differt.*

#### Type.

**Brazil**. Bahia: Mun. Jequié, Distrito de Cachoeirinhas, caatinga arbustiva em topo de morro, com lajeados graníticos, 299 m, 13°54'14.4"S, 40°01'46.8"W, 10 Jul 2009 (fr), *L.L. Giacomin 974* (holotype: BHCB; isotypes, BM, MBM, NY, RB).

#### Description.

Herbs, slightly woody to woody at base, few- to many-branched, 20–40 cm tall. Stems moderately to densely pubescent with multicelled unbranched erect glandular hairs ca. 0.3–0.5 mm long, these mixed with less frequent slightly longer 1–3-celled unbranched eglandular hairs. Sympodial units difoliate, solitary or more commonly geminate, the smaller leaves up to half the size of the larger ones. Leaves simple, the blades 1–4 *×* 1–3 cm, ovate-elliptic to cordiform, chartaceous to membranaceous, sparsely to moderately pubescent on both sides with 1–2-celled unbranched erect eglandular hairs, these denser on the primary and secondary veins; venation camptodromous, with the primary and one pair of secondary veins emerging from the leaf base (sometimes just one, in the case of an asymmetric base), the primary and secondary veins barely visible to the naked eye, slightly prominent abaxially and less visible adaxially; base attenuate to cordate, slightly decurrent into petiole; margins entire, ciliate with hairs like those of the blade; apex acute to attenuate; petioles 0.5–2.2 cm long, with pubescence similar to that of the stems but with fewer eglandular hairs. Inflorescencessessile, lateral, extra-axillary or subopposite the leaves, unbranched, with 1–4 flowers, the axes with pubescence like that of the stems; peduncles absent; rachis very short; pedicels 6–10 mm in flower, 7–14 mm in fruit, almost contiguous, articulated at the base. Flowers 5-merous. Calyx 2–7 mm long, the tube 1–2 mm, the lobes 2–6 *×* 1–2.6 mm, ovate-elliptic, the apex acuminate, moderately pubescent abaxially with almost exclusively glandular unbranched multicellular erect hairs, densely pubescent adaxially with very small glandular hairs with 1-celled stalks; calyx accrescent in fruit, the lobes up to 8 mm long, equal to or exceeding the berry at maturity. Corolla 1–2.5 (-3) cm in diameter, rotate with abundant interpetalar tissue, membranaceous, white, the lobes 2–4 *×* 1–3 mm, triangular, acute at apex, with a few eglandular hairs abaxially, mainly on the central part of each lobe, glabrous adaxially. Stamens 4–9.5 mm long; filaments 1–2 mm long, with one much longer than the others, up to 5 mm long, glabrous; anthers 4–6 *×* 1.3–2 mm, connivent, yellow, the base cordate, with a small bulge dorsally, the apex emarginate, the pores directed introrsely and subapically, not opening into longitudinal slits. Ovary glabrous; style 7–9 mm long, longer than the smaller stamens, cylindrical, glabrous, curved near apex, closely appressed to the larger stamen; stigma capitate. Fruits 0.8–1.5 cm in diameter, globose berries, greenish white when immature, translucent at maturity, drying light-brown to blackish, glabrous, the mesocarp watery and held under pressure, dehiscing explosively at maturity, normally between two calyx lobes**.** Seeds 10–25 per fruit, 2.5–3.6 *×* 1.8–2.9 mm, flattened, reniform, with a small hollow where connected to the placenta, the margin flattened, forming a winged projection, the seed surface with raised projections and grooves parallel to margin, giving a netlike impression.

**Figure 1.**
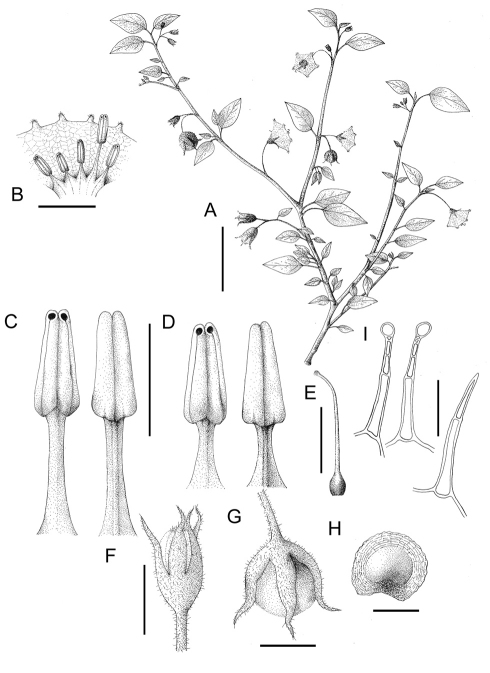
*Solanum evolvuloides* Giacomin & Stehmann. **A** Habit **B** Corolla cross section showing stamens **C** Larger stamen in ventral and dorsal view **D** Smaller stamens in ventral and dorsal view **E** Gynoecium **F** Young bud **G** Fruit **H** Seed **I** Detail of the hairs of stems, peduncles and calyces (glandular) and leaves (eglandular). All from *Mattos-Silva* *s.n.* (CEPEC-15698). Scale bars A= 3 cm; B, C, D, E, F and G = 5 mm; H = 2 mm ; I = 0.2 mm.

**Figure 2.**
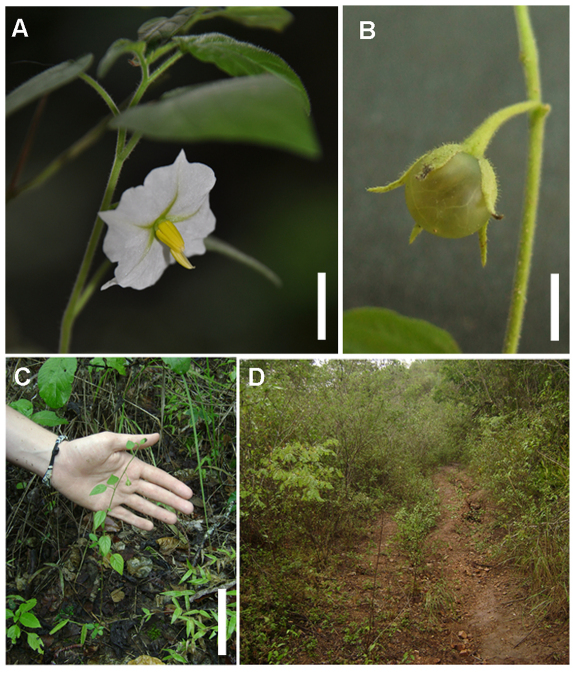
*Solanum evolvuloides* Giacomin & Stehmann. **A** Flower, opened during the morning in a cultivated seedling **B** Fruit, with a semi-transparent exocarp **C** Habit **D** Habitat in a shrubby caatinga formation. Scale bars A= 1.5 cm, B= 1 cm; C = 10 cm. All from Giacomin 974 (BHCB; type collection).

**Figure 3.**
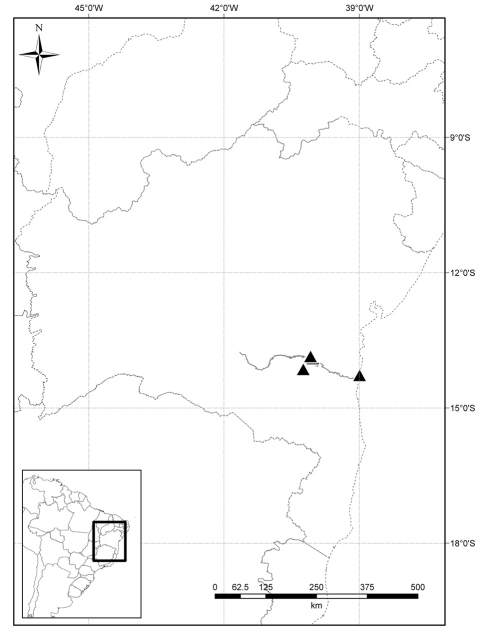
Known distribution of *Solanum evolvuloides* Giacomin & Stehmann (triangles) along Rio de Contas (in grey) in the state of Bahia, Brazil.

**Distribution.** This species is known only from the southeastern part of Bahia state (Fig. 3), Brazil, occurring in the transition zone between deciduous forests and xeric formations of shrubby Caatinga (as defined by Velloso et al. 2002).

**Ecology.** *Solanum evolvuloides*was recently recollected by the first author in the municipality of Jequié in a typical shrubby Caatinga formation, that is associated in this region with large granitic outcrops. The occurrence on the banks of the Rio de Contas near the city of Itacaré [*Jardim, J.G. 1843* (CEPEC)] might be an occasional case of water dispersal by the river, which arises in a xeric environment near the center of the state in the Caatinga biome. Despite having been found in environments with marked seasonality, the species is apparently not annual, as evidenced by the woody stem bases.

**Etymology.** The name *Solanum evolvuloides* evokes the strong resemblance of this species to a sympatric species of the genus *Evolvulus* (Convolvulaceae).

**Phenology.** Flowering and fruiting material has been collected between February and August, with a flowering peak from February to May; fruiting specimens were collected from June to August. Under cultivation, flowers were observed to open only in the morning, closing during the evening. Observations of the same flower during consecutive days confirmed this pattern.

**Preliminary conservation status.** Endangered (EN) B1 a,b (i,ii,iii,iv). *Solanum evolvuloides* is known from only two localities, where the landscape has been strongly modified in the last decades due to the expansion of urban centers and extensive farming. The region has been focus of several surveys undertaken by the CEPEC group, in association with the New York Botanical Garden; despite this, only a few collections of this species have been made. Although one collection was made in a very disturbed area (*Jardim 1843*), the most recent collection is from a well-preserved forest fragment, and the species was not found in surrounding areas. There are no collections from within conservation units.

**Specimens examined (Paratypes).** BRAZIL. Bahia: Mun. Itacaré. Fazenda Monte Alegre, Ca. de 1 km a leste na rodovia para Itacaré. Margem do Rio de Contas, 10 August 1998 (fl, fr), J.G. Jardim 1843 (CEPEC); Mun. Jequié. Rodovia Ipiaú/Jequié, 12 May 1969 (fl, fr), J.A. Jesus 367 (CEPEC); Mun. Jequié. Km 7 da estrada Jequié/Ipiaú, Caatinga, 10 February 1983 (bs), A.M. Carvalho, 1591 (CEPEC); Mun. Jequié, Procedente do Distrito de Cachoeirinhas, caatinga arbustiva em topo de morro, com lajeados graníticos, 299 m, 13°54'14.4"S, 40°01'46.8"W, floresceu em cultivo no Jardim Botânico da Fundação Zoo-Botânica de Belo Horizonte, 23 September 2009 (fl), *L.L. Giacomin 974B* (BHCB, NY); Mun. Manoel Vitorino. Rod. Man. Vitorino / Caatingal. Km 4. região de Caatinga. 16 February 1979 (fl, fr), L.A. Mattos Silva s.n. (CEPEC [15698]).

**Discussion.** The species is similar to *Solanum turneroides* Chodatand they are the only species within the section presenting strong heterandry, with one stamen with a filament much longer than the other four. Sometimes *Solanum parcistrigosum* Bitterand *Solanum hoffmannseggii* Sendtner, species that also resemble *Solanum evolvuloides*, are weakly heterandrous but they both have smaller flowers (corolla with < 1.5 cm in diameter) and stamens, and can be easily distinguished from *Solanum evolvuloides* by the glandular indument observed in the calyx and stems of the latter species. This character can be also used to separate *Solanum evolvuloides* from *Solanum turneroides*. These two speciesare also not sympatric, in Brazil *Solanum turneroides* is found only in the states of Mato Grosso do Sul and São Paulo, whereas *Solanum evolvuloides* occurs only in Bahia. *Solanum turneroides* has an indument composed of unbranched eglandular hairs mainly with one-celled appressed hairs on the calyces, leaves and stems, associated with two-celled hairs that are typically geniculate (bent at a 90° angle) between the first and second cell. It is also a more robust shrub with chartaceous leaves, in contrast to the more membranaceous leaves of *Solanum evolvuloides*.

Within the section, glandular hairs are commonly found on the leaves of *Solanum adscendens* Sendtner but in this species, the hairs are much smaller (up to 0.1 mm, barely visible in dried material) than those of *Solanum evolvuloides*, have glands composed of more than one cell, and are associated with several erect eglandular hairs. The hairs of *Solanum evolvuloides*, perhaps the most distinctive characteristic of the species, are up to 0.5 mm long, are stalked, have unicellular glands, and are not commonly found on the leaves.

The heterandry found in *Solanum evolvuloides* is not a common feature within *Solanum*, and its evolution had been focus of recent studies, using morphological or molecular data (Lester et al. 1999, Bohs et al. 2007). Both cited works conclude that this character evolved several times independently within the genus. Within the Brevantherum clade (*sensu* Weese and Bohs 2007), only the two species of sect. *Gonatotrichum* cited above are known to be strongly heterandrous. The species do not present strong enantiostyly, but in both cases a deflection on the style apex is observed (see Fig. 2A), that might function to receive pollen from a bee’s abdomen, as pointed out in other studies of heterandrous and enantiostylous *Solanum* species (e.g. Vallejo-Marín 2009).

## Supplementary Material

XML Treatment for Solanum evolvuloides
